# Hyperprogressive Disease Caused by PD-1 Inhibitors for the Treatment of Pan-Cancer

**DOI:** 10.1155/2021/6639366

**Published:** 2021-06-22

**Authors:** Shiyun Chen, Miaomiao Gou, Huan Yan, Mengjiao Fan, Yuting Pan, Runjia Fan, Niansong Qian, Guanghai Dai

**Affiliations:** ^1^Medical School of Chinese PLA, Beijing 100853, China; ^2^Department of Oncology, Chinese PLA General Hospital, Beijing 100853, China; ^3^The Second School of Clinical Medicine, Southern Medical University, Guangzhou, 510220 Guangdong Province, China; ^4^The Hainan Medical Center, Chinese PLA General Hospital, Sanya 572000, China

## Abstract

**Background:**

Nowadays, PD-1/PD-L1 inhibitors are widely used to treat various malignant tumors. However, during the immunotherapy in a few patients, a flare-up of tumor growth occurred. This new pattern of progression is called hyperprogressive disease (HPD). *Patients and Methods*. The retrospective study included 377 patients with various malignant tumors treated with PD-1 inhibitors (nivolumab or pembrolizumab) in the Chinese PLA General Hospital from January 2015 to January 2019. Clinicopathologic variables, tumor growth rate (TGR), and treatment outcomes were analyzed in patients with pan-cancer treated with PD-1 inhibitors. HPD was defined as the difference of TGR before and during immunotherapy exceeding 50%.

**Results:**

In 38 of 377 patients (10.08%), HPD occurred after treatment with PD-1 inhibitors. Patients with HPD had lower overall survival (OS) than those without HPD (median OS, 3.6months (95% CI, 3.0–4.2) vs. 7.3 months (95% CI, 5.9–8.7); *P* < 0.01). Factors related to HPD include more than 2 metastatic sites, ECOG performance status ≥ 2, hepatic metastases, and lactate dehydrogenase level greater than normal upper limit. KRAS status was significantly associated with HPD in patients with colorectal cancer. In the exploratory predictors' analysis, the rapid increase of characteristic tumor markers (such as CEA in colorectal cancer, CA199 in pancreatic cancer and cholangiocarcinoma) within one month was found to be associated with the occurrence of HPD.

**Conclusions:**

HPD was developed with different rates in a variety of malignant tumor patients treated with PD-1 inhibitors and related to some clinicopathological features and poor prognosis. Tumor markers, especially CA199, might be served as early predictors of HPD.

## 1. Introduction

The interaction between the human immune system and tumor growth has been a hot topic in recent years. The PD-1/PD-L1 pathway plays a crucial role in the immune evasion process of tumors. Immune checkpoint inhibitors, PD-1/PD-L1 inhibitors, have been applied to the treatment of a variety of malignant tumors including non-small-cell lung cancer (NSCLC) [1–3], melanoma [4–6], head and neck squamous cell carcinoma (HNSCC) [7, 8], and metastatic renal cell cancer [9] and have become the standard treatment for some cancers, which are pretty promising. However, tumor progression was accelerated for some patients after treatment with PD-1/PD-L1 inhibitors. This new pattern of progression is called hyperprogressive disease (HPD), and the rates range from 4% to 29% [10]. At present, there is no uniform definition of HPD, and its possible predictive biomarkers are still being explored. However, the occurrence of HPD during immunotherapy caused a significant decrease in the overall survival (OS) of the patient, which seriously affected the treatment effect [11, 12]. In order to avoid the potential risks during the treatment with PD-1/PD-L1 inhibitors, it is necessary to actively explore the clinicopathological features and biomarkers related to the occurrence of HPD. In this study, the incidence of HPD was evaluated by calculating tumor growth kinetics (TGK) before and during treatment with PD-1 inhibitors (nivolumab/pembrolizumab) in patients with various types of malignant tumors, and their associated clinicopathological characteristics were analyzed to explore its possible predictors.

## 2. Materials and Methods

### 2.1. Patients

The data from consecutive eligible patients with various types of stage IV malignant tumors treated with PD-1 inhibitors (nivolumab/pembrolizumab) from January 2015 to January 2019 from the Chinese PLA General Hospital were studied retrospectively. Inclusion criteria for patients are as follows: (1) 18 years old and above, (2) patients with stage IV malignancy confirmed by histological, (3) available CT scans before and after immunotherapy, (4) received PD-1 inhibitor therapy, and (5) had measurable target lesions judged based on RECIST v1.1 (Response Evaluation Criteria in Solid Tumors). A total of 377 patients were included in the final analysis after screening. The patients with pseudoprogression were excluded in the HPD group. Data on the following variables were collected to investigate the relationship between them and the occurrence of HPD in patients with malignant tumors treated with PD-1 inhibitors: age, smoking history, malignant tumor type, surgery history, PD-L1 expression on tumor cells, response to line before treatment with PD-1 inhibitors, number of metastatic sites, method of medication (monotherapy or combination), Eastern Cooperative Oncology Group (ECOG) performance status, lactate dehydrogenase (LDH) levels, liver metastasis, and neutrophil-to-lymphocyte ratio. In addition, the KRAS status in patients with NSCLC and colorectal cancer were collected, but EGFR status was collected only in patients with NSCLC.

### 2.2. Definition of Tumor Growth Rate (TGR) and HPD

CT scans for at least 3 time points were needed: CT scans 6-8 weeks before baseline, CT scans at baseline (when PD-1 inhibitors started), and CT scans 6-8 weeks after baseline and at least 4 weeks between CT scans. TGR was defined as the percentage increase in tumor volume per month. TGR was calculated only with measurable target lesions, and based on the sum of the longest diameter of the target lesion described in the RECIST 1.1 version and the definition by Ferté and colleagues (See Document S1 in the Supplementary Material) [13], new lesions were not included. TGR from 6-8 weeks before baseline to baseline was set to TGR^PRE^, similarly, TGR from baseline to 6-8 weeks after baseline was set to TGR^POST^, and difference of TGR was defined as TGR^D^ = TGR^POST^ − TGR^PRE^. The target lesions in patients whose response was assessed as progressive disease (PD) between 6 and 8 weeks after treatment with the PD-1 inhibitors were reevaluated; tumor volume variation was calculated. HPD was defined as the difference between TGR^POST^ and TGR^PRE^ more than 50% (TGR^D^ ≥ 50%).

### 2.3. Statistical Analysis

*χ* ^2^ or Fisher's exact test was used for the categorical variables. The cut-off values of tumor markers' increase percentage were determined with receiver operating characteristic (ROC) curves. The OS curves were depicted by the Kaplan-Meier method and compared with a log-rank test. All tests were two-tailed; it was considered statistically significant if the value of *P* was less than 0.05. Statistical analyses were performed using SPSS version 23.

## 3. Results

### 3.1. Patient Characteristics

A total of 698 patients with the treatment of PD-1 inhibitors (nivolumab/pembrolizumab) from January 2015 to January 2019 from the PLA General Hospital were included in the initial database. According to the inclusion and exclusion criteria, a total of 377 patients were included in the final characteristic analysis after screening (Table 1). Most of the patients were younger than 60 years (57.6%) and had relatively good performance status (ECOG 0 or 1: 95.5%). In this study, the patients with lung cancer (in total, 35.3%) accounted for the largest proportion among all types of malignant tumors. Most patients (327 out of 377) carried out PD-L1 immunohistochemistry and about two-thirds were positive (205/327, 62.7%).

**Figure 1:**
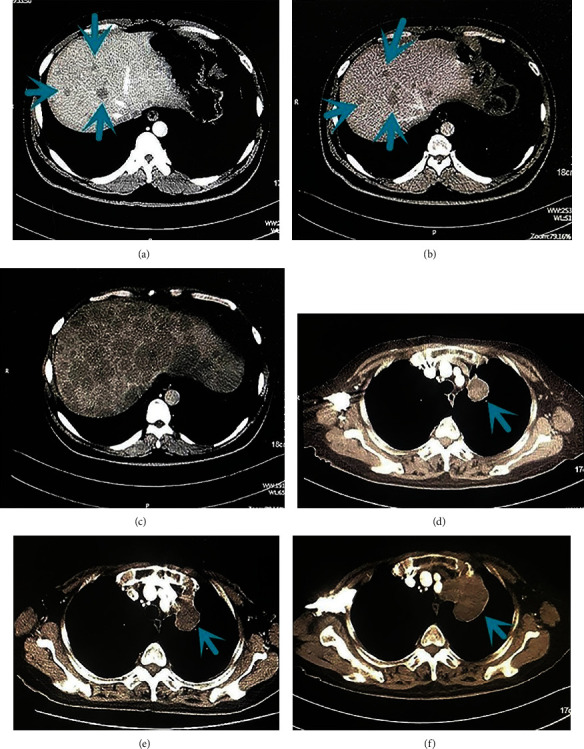
Computed tomographic scans before baseline (a), at baseline about 6-8 weeks later (b), and during PD-1 and programmed death ligand 1 (PD-L1) inhibitor therapy 6-8 weeks later (c) in a man in his mid-40s with stage IV (mediastinal lymph nodes, right adrenal gland, and liver metastases) non-small-cell lung cancer treated with anti-PD-1 and anti-PD-L1 therapy in the sixth line. After 2 administrations, there was evidence of significant liver lesion progression. Arrowheads show liver lesions before and during anti-PD-1+anti-PD-L1 treatment. Computed tomographic scans before baseline (d), at baseline about 6-8 weeks later (e), and during programmed death 1 (PD-1) inhibitor therapy 6-8 weeks later (f) in a woman in her mid-70s with stage IV (hilar and mediastinal lymph nodes and jejunum metastases) EGFR L858R missense mutation and TP53 mutation lung adenocarcinoma treated with anti-PD-1 therapy in the third line. After 2 administrations, there was evidence of significant lung progression. Arrowheads show lung lesions before and during anti-PD-1 treatment.

**Figure 2:**
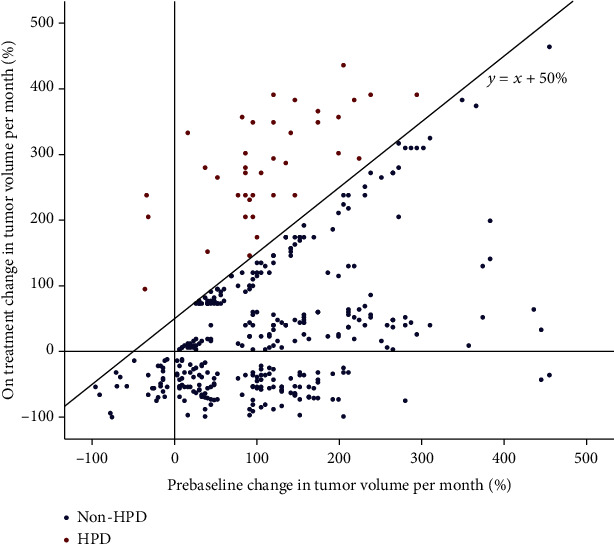
Blue spots show 339 patients with nonhyperprogressing disease (non-HPD). Red spots show 38 patients with hyperprogressive disease (HPD) during PD-1 inhibitor therapy.

According to RECIST 1.1 evaluation, the best response of 125 (33.2%) patients after immunotherapy was PD, and HPD was observed in 38 patients (10.1%) (Figures 1 and 2) after we excluded the patients with pseudoprogression.

### 3.2. Clinicopathologic Factors Associated with HPD

After treatment with PD-1 inhibitor, HPD was more frequently observed in the patients with more metastatic sites (*P* < 0.01), liver metastasis (*P* < 0.01), ECOG score ≥ 2 (*P* < 0.01), and LDH levels higher than normal (*P* = 0.028) before immunotherapy. In addition, it was observed that patients with PD (*P* = 0.034) or some types of tumor (e.g., esophageal cancer, colorectal cancer, and pancreatic cancer) (*P* < 0.01) (Tables 1 and 2) and patients with colorectal cancer who had KRAS mutations (*P* = 0.039) (Table 3) were more likely to have HDP after subsequent immunotherapy.

### 3.3. Association between HPD and the Increase of Tumor Markers

**Figure 3:**
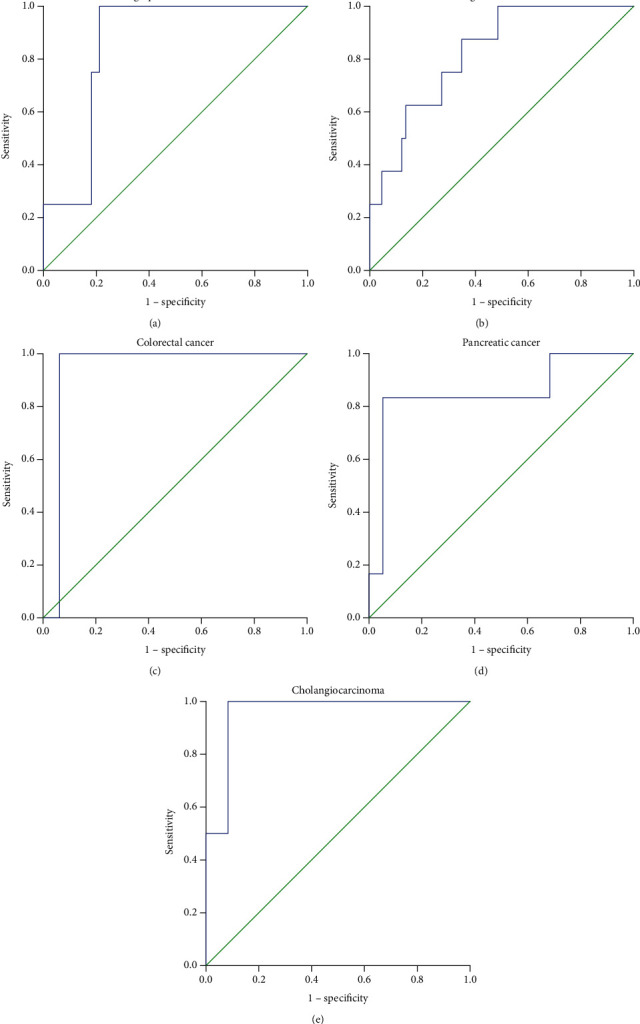
Receiver operating characteristic (ROC) curves for major tumor biomarkers of various types of tumors ((a) lung squamous carcinoma, (b) lung adenocarcinoma, (c) colorectal cancer, (d) pancreatic cancer, and (e) cholangiocarcinoma) compared with the evaluation results of response evaluation criteria in solid tumor (RECIST) standards and HPD standard in this study to determine the cut-off values for the occurrence of HPD.

It was found that after the treatment with PD-1 inhibitors, the increase of tumor markers may have an indicative effect on the development of HPD. The serum level changes of characteristic tumor markers (such as CEA in colon cancer, AFP in liver cancer, and PSA in prostate cancer) of various types of tumors after the treatment with PD-1 inhibitors were compared with the evaluation results of RECIST standard and HPD standard in this study. Only the cancer types with statistical significance were listed in the figures and tables below (Figure 3 and Tables 4 and 5). According to the ROC curve (*P* < 0.05), the area under curve (AUC) was 0.856 for lung squamous carcinoma, 0.824 for lung adenocarcinoma, 0.938 for colorectal cancer, 0.857 for pancreatic cancer, and 0.958 for cholangiocarcinoma. Before the radiological evaluation, if the serum tumor markers CYFRA211/NSE/SCC increased by 12% for lung squamous carcinoma, CEA/CA125 increased by 29% for lung adenocarcinoma, CA125/CA724/CA199/CEA increased by 49% for colorectal cancer, CA199 increased by 167% for pancreatic cancer, and CA199 increased by 126% for cholangiocarcinoma accordingly per month from the baseline, the possibility of HPD of the patient was considered to be high. At this point, a largest Youden index was provided (Table 4). In addition, patients with pancreatic cancer had the highest cut-off value of tumor marker (CA199), which reached 167%, and it might be the most indicative of HPD. Therefore, the positive predictive value and negative predictive value of patients with pancreatic cancer were counted separately, which were 85.7% and 94.4%, respectively (Table 5).

### 3.4. Survival Data

**Figure 4:**
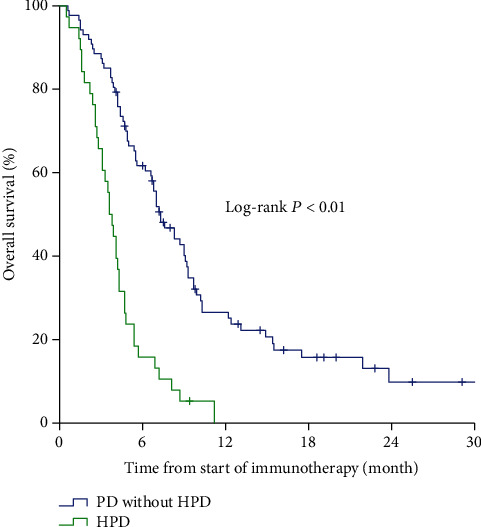
Overall survival for HPD compared with progressive disease (PD) without hyperprogression in patients with immunotherapy.

Based on the survival analysis of the patients with HPD and PD patients without HPD, OS of patients with HPD was significantly lower (median OS, 3.6 months (95% CI, 3.0-4.2 months) vs. 7.3 months (95% CI, 5.9–8.7months); *P* < 0.01) (Figure 4).

### 3.5. Association between HPD and Therapy Mode

In addition, the data of the patients treated with PD-1 inhibitor monotherapy, dual-immunotherapy (PD-1 inhibitors + CTLA-4 inhibitors), and combination therapy (PD-1 inhibitors+chemotherapy/targeted therapy) were used to analyze their difference of HPD incidence, which was 12.9%, 25.0%, and 8.0%, respectively. But the difference of the HPD incidence was not statistically significant (*P* = 0.12) (Table 6).

## 4. Discussion

To our knowledge, most of the studies on HPD induced by immunotherapy had only focused on monocarcinoma. Previously, Champiat et al. had studied on HPD caused by immunotherapy for pan-cancer [14], and our study is one of the few studies on pan-cancer. This study included multiple types of malignancies, HPD occurred in 10.1% (38/377) of patients treated with PD-1 inhibitors. In previous studies about immunotherapy for lung cancer, it was reported that HPD occurred in 13.8% of patients due to the treatment of PD-1/PD-L1 inhibitors by Ferrara et al. [11], while Kim et al. reported an incidence of HPD with approximately 19% [15]. In addition, in another study of 34 patients with recurrent and/or metastatic head and neck squamous cell carcinoma, the incidence of HPD after the treatment with PD-1/PD-L1 inhibitors was 29% [16], which is the highest reported incidence known at the moment. This difference in incidence might be due to tumor types or their different definitions of HPD. Until now, there are not many studies on HPD, so the incidence of HPD after immunotherapy for many types of tumors is largely unknown.

In this study, the largest proportion of the patients was lung cancer patients, and the incidence of HPD was 10.53%, which was closer to some previous related reports [11, 17]. In addition, it was found that the incidence of HPD varied according to the type of cancer, and it was statistically significant. The tumors with relatively high incidence of HPD were pancreatic cancer (28.0%), colorectal cancer (22.7%), and esophageal cancer (20.0%). Most of our statistics were based on the patients in the medical department of oncology treated with PD-1 inhibitors. The exact definition of HPD was inconclusive at present. Champiat et al. defined that first evaluation was assessed as progression according to RECIST 1.1, and TGR during experimental periods was more than twice as much as reference periods [14].

Ferrara et al. believed that based on the first evaluation of disease progression, HPD was defined as *Δ*TGR (TGR during immunotherapy minus TGR before treatment) exceeding 50% per month [11]. And Sasaki et al. described HPD as the change of the sum of the largest diameters of target lesions per unit time during treatment with PD-1/PD-L1 inhibitors, compared with TGK before treatment; TGK increased at least twofold [18]. Similar to the definition of HPD used in the study of Ferrara et al., in our study, TGR was also calculated based on the volume change of the target lesion per unit time to infer whether HPD has occurred in patients, because we hold the opinion that evaluating tumor changes based on tumor volume is more rigorous than based on tumor diameter. For example, for a tumor with a volume increase of 50% per month before immunotherapy, it was evaluated as HPD, only when the tumor increased at least 100% monthly during the immunotherapy. However, because at least 3 consecutive CT scans were required within the specified time, many patients who did not meet this requirement were excluded, leading to an underestimation of the incidence of HPD.

Previous studies reported that HPD could lead to a reduction in the OS of patients [11, 15]. In this study, it was also found that HPD occurring in the patients treated with PD-1 inhibitor was significantly associated with poor OS, so it was important to find the predictive biomarkers. It might have a predictive value for the occurrence of HPD, if the major tumor biomarkers of various types of tumors (such as CA199 commonly used in pancreatic cancer and cholangiocarcinoma and CEA commonly used in colorectal cancer) increased above a certain level within 1 month after immunotherapy (before radiological evaluation). In Tables 4 and 5 of this study, only the cancers with statistical significance were listed, while the other cancers were not statistically significant, which could be caused by insufficient data. If the changes of characteristic tumor markers are used to predict HPD in each cancer type, their cut-off values are greatly different. For lung squamous carcinoma, the cut-off value was only 12%, while for pancreatic cancer, the cut-off value was as high as 167%. It was considered that perhaps tumor markers were not so sensitive to predict the occurrence of HPD in lung squamous carcinoma. After statistical analysis of the positive predictive value and negative predictive value, it was found that CA199 had a very important predictive effect on the occurrence of HPD in patients with pancreatic cancer. This meant that in clinical practice, if the tumor biomarkers of patients treated with PD-1 inhibitors increase significantly in the short term, it is necessary to be more cautious to continue the subsequent treatment. If necessary, radiological evaluation can be performed in advance and combined with the patient's clinical symptoms to evaluate the situation.

Recent studies have shown that derived neutrophil-to-lymphocyte ratio was associated with HPD and was an independent predictor of OS [19]. But it was not found in our analysis. This might be due to the insufficient amount of data and the less quantity of patients with HPD. Our study found that the incidence of HPD in subsequent immunotherapy was relatively low in those patients who had relatively good curative effect in the previous treatment. Before immunotherapy, the response to line was PD, the number of metastatic sites was more than 2, the ECOG performance status was greater than 2, and LDH level was higher than the normal upper limit or liver metastases; these mentioned factors were indicators that reflected the overall state of the patient and tumor burden and related to the occurrence of HPD after subsequent treatment with PD-1 inhibitors; therefore, HPD might be more likely to occur in the patients with worse baseline status before immunotherapy, and some of these findings were consistent with previous research reports [11, 15]. In addition, there were reports that KRAS-driven lung cancer often inactivated STK11/LKB1 and responded poorly to immunotherapy [20]. Recent studies have shown that the simultaneous occurrence of STK11 and KRAS mutations could be used as a potential biomarker for HPD prediction [17]. In this study, KRAS status was collected only in patients with NSCLC (see Table S1 in the Supplementary Material) and colorectal cancer, statistical significance was found in patients with colorectal cancer. It was more likely to developed HPD for patients with KRAS mutations after treatment with PD-1 inhibitors. However, due to the small number of colorectal cancer patients, this result has certain limitations. In the future, the sample size needs to be further expanded. In addition, we did not draw this conclusion in patients with NSCLC, which might be related to some missing data of our genetic testing.

After statistical analysis, it was found that the incidence of HPD in patients with PD-1 inhibitor monotherapy, dual-immunotherapy, and immunotherapy combined with other treatments (chemotherapy/targeted therapy) was 12.9% (17 of 132), 25.0% (2 of 8), and 8.0% (19 of 237), respectively, but no statistical difference was found. This meant, the possibility of HPD existed in both monotherapy and combination therapy including PD-1 inhibitors. However, the issues whether incidence of HPD will be aggravated in dual-immunotherapy and be hindered with additional chemotherapy cannot be determined for the time being and needs to be resolved in future studies.

Our study had some limitations, mainly because it was a retrospective study in single institution with a limited sample size. It was impossible to obtain genetic testing results for all patients. Because it was not routinely assessed in clinical practice, a large amount of relevant data was missing, so that we could not accurately describe the interaction between a genetic mutation and HPD. At least three consecutive CT scans within a specified time were required in our screening conditions, so many patients were excluded. In addition, we only focused on the changes of the target lesions and did not evaluate nontarget lesions (such as pleural effusion and ascites), resulting in the inaccurate estimation of the incidence of HPD. Finally, although there were a large number of samples in this study, due to the nature of retrospective study, it is impossible to explore the mechanism of HPD in depth. It is currently believed that the mechanism of HPD after treatment with PD-1/PD-L1 inhibitors might be related to the increase in the quantity of Treg cells, the exhaustion of T cells, the polarization of the affected immunosuppressive cell subsets, the trigger of uncontrolled inflammation, and the activation of protooncogene signaling pathways/gene mutations, etc. [12]. In addition, the relationship between MDM2 family amplification or EGFR aberrations and the occurrence of HPD also remains to be further research [21]. In the future, dynamic monitoring and evaluation of tumor tissue or blood before and during treatment with PD-1 inhibitors can be performed. This type of prospective study will be helpful to determine biomarkers and the occurrence mechanism of HPD.

## 5. Conclusions

This study shows that HPD could lead to a severely poor prognosis with a negative impact on OS. Among patients with immunotherapy, the incidence of HPD was different for various types of tumors, and it was related to certain clinicopathological and molecular characteristics. In addition, the results suggested that monitoring changes in tumor markers during PD-1 blockade has clinical implications in predicting HPD. However, further studies are needed to clarify the molecular mechanism of HPD and its biomarkers.

## Figures and Tables

**Table 1:** Association between clinical categorical variables and HPD for immunotherapy-treated patients (n, %).

Variable	Non-HPD (*n* = 339)	HPD (*n* = 38)	*P*
Age (y)			0.696
≥60	145 (42.8)	15 (39.5)	
<60	194 (57.2)	23 (60.5)	
Smoking history			0.091
Ever-smoker	138 (40.9)	21 (55.3)	
Never-smoker	199 (59.1)	17 (44.7)	
Missing	2	0	
Smoking exposure (pack-years)			0.737
>30	89 (26.4)	11 (28.9)	
≤30	248 (73.6)	27 (71.1)	
Missing	2		
Surgical history			0.800
Yes	159 (46.9)	17 (44.7)	
No	180 (53.1)	21 (55.3)	
PD-L1 positivity			0.173
Positive	185 (64.0)	20 (52.6)	
Negative	104 (36.0)	18 (47.4)	
Missing	50	0	
Response to line before immunotherapy			0.034
PR	9 (2.6)	0 (0.0)	
SD	125 (36.9)	7 (18.4)	
PD	205 (60.5)	31 (81.6)	
Number of metastatic sites			0.000
0-1	227 (67.0)	14 (36.8)	
≥2	112 (33.0)	24 (63.2)	
ECOG performance status			0.007
≥2	12 (3.5)	5 (13.2)	
0-1	327 (96.5)	33 (86.8)	
Neutrophil-to-lymphocyte ratio			0.726
>3	124 (36.6)	15 (39.5)	
≤3	215 (63.4)	23 (60.5)	
Lactate dehydrogenase level			0.028
≤upper limit of normal	222 (65.5)	18 (47.4)	
>upper limit of normal	117 (34.5)	20 (52.6)	
Liver metastasis			0.000
Present	97 (28.6)	24 (63.2)	
Absent	242 (71.4)	14 (36.8)	

HPD: hyperprogressive disease; PD-L1: programmed cell death 1 ligand 1; PR: partial response; SD: stable disease; PD: progressive disease; ECOG: Eastern Cooperative Oncology Group.

**Table 2:** Association between tumor types and HPD for Immunotherapy-treated patients.

Tumor types	Non-HPD (*n* = 339)	HPD (*n* = 38)	The incidence of HPD	*P*
Lung cancer	119	14	10.53%	
Pancreatic cancer	18	7	28.00%	
Esophageal cancer	8	2	20.00%	
Colorectal cancer	17	5	22.73%	
Cholangiocarcinoma	19	3	13.64%	
Liver cancer	15	2	11.76%	<0.01
Lymphoma	25	1	3.85%	
Ampullary carcinoma	5	1	16.67%	
Cervical carcinoma	6	1	14.29%	
Gastric carcinoma	31	2	6.06%	
Other	76	0	0.00%	

HPD: hyperprogressive disease.

**Table 3:** Association between the KRAS status and HPD for patients with colorectal cancer treated with immunotherapy (*n*, %).

Colorectal cancer	Non-HPD (*n* = 17)	HPD (*n* = 5)	*P*
KRAS mutation			0.039
Mutated type	4 (23.5)	4 (80.0)	
Wild type	13 (76.5)	1 (20.0)	

HPD: hyperprogressive disease.

**Table 4:** HPD according to ROC curves.

Tumor types	Characteristic tumor markers	Cut-off values	Sensitivity	Specificity	*P* value
Lung squamous carcinoma	CYFRA211/NSE/SCC	12%	100.0%	78.8%	0.022
Lung adenocarcinoma	CEA/CA125	29%	87.5%	65.2%	<0.01
Colorectal cancer	CA125/CA724/CA199/CEA	49%	100.0%	93.7%	<0.01
Pancreatic cancer	CA199	167%	85.7%	94.1%	<0.01
Cholangiocarcinoma	CA199	126%	100.0%	91.7%	0.034

HPD: hyperprogressive disease; ROC: receiver operating characteristic.

**Table 5:** Positive predictive value and negative predictive value of HPD in patients with pancreatic cancer with immunotherapy according to CA199 test when the cut-off value is 167%.

Evaluation parameter	CA19-9
Sensitivity	85.7%
Specificity	94.1%
Positive predictive value	6/7 (85.7%)
Negative predictive value	17/18 (94.4%)

HPD: hyperprogressive disease.

**Table 6:** Association between HPD and single or combination immunotherapy.

Monotherapy or combination	Total (*N* = 377)	Non-HPD (*N* = 339)	HPD (*N* = 38)	*P* value
Monotherapy	132	115	17	0.121
Dual-immunotherapy	8	6	2	
Combine with chemotherapy/targeted therapy	237	218	19	

HPD: hyperprogressive disease.

## Data Availability

(1) The data is a clinical statistic data. (2) The (clinical statistic) data used to support the findings of this study are available from the corresponding author upon request. (3) The above data are the foreshadowing of our next research, so requests for access to these data should be made to Shiyun Chen (chenshiyungl@163.com) or Niansong Qian (kyotomed@foxmail.com).

## Supplementary Materials

Supplementary Materials Supplementary 1. Document S1: calculation method of tumor growth rate. Supplementary 2. Table S1: association between the genetic status and HPD for patients with non-small-cell lung cancer treated with immunotherapy.
