# Supplementary material for: Hyperprogressive Disease Caused by PD-1 Inhibitors for the Treatment of Pan-Cancer
Source: Dis Markers. 2021 Jun 22;2021:6639366. doi: 10.1155/2021/6639366 (PMC8241516; doi:10.1155/2021/6639366)
Supplement: Supplementary Materials — Supplementary 1. Document S1: calculation method of tumor growth rate. Supplementary 2. Table S1: association between the genetic status and HPD for patients with non-small-cell lung cancer treated with immunotherapy. [file 6639366.f1.docx]

*Supplementary 1.* Document S1: Calculation method of tumor growth rate

According to the RECIST criteria, *t* is defined expressed in months at the tumor evaluation, and *D*_t_ is defined as the sum of the longest diameters of the target lesions. Assuming that the tumor growth follows the exponential law, the tumor volume at time *t* is:

$$V_{t}=V_{0}e^{TG*t}$$

$\frac{V_{t}}{V_{0}}=e^{TG*t}$ (1-1)

Where *V*_0_ represents the tumor volume at baseline and *TG* represents the growth rate.

Tumor volume can also be expressed by the following formula:

$$V_{t}=\frac{4}{3}\pi{R_{t}}^{3}=\frac{4}{3}\pi{(\frac{D_{t}}{2})}^{3}$$

$$V_{0}=\frac{4}{3}\pi{R_{0}}^{3}=\frac{4}{3}\pi{(\frac{D_{0}}{2})}^{3}$$

$\frac{V_{t}}{V_{0}}={(\frac{D_{t}}{D_{0}})}^{3}$ (1-2)

*TG* results from the logarithm to the base of the mathematical constant *e* of 1-1 and 1-2:

$$\ln e^{TG*t}=\ln{(\frac{D_{t}}{D_{0}})}^{3}$$

$$TG*t=3\ln\frac{D_{t}}{D_{0}}$$

$$TG=\frac{3\ln\frac{D_{t}}{D_{0}}}{t}$$

*TGR* is defined as a percent increase in tumor volume per month:

$$TGR=100 \frac{V_{t}-V_{0}}{V_{0}}=100 \frac{V_{0}e^{TG}-V_{0}}{V_{0}}=100 (e^{TG}-1)$$

Tumor volume was evaluated at three timepoints: before treatment with Immune Checkpoint Inhibitor [(n-1) CT scan], at baseline [baseline (n) CT scan], and once during treatment [(n+1) CT scan]. *TGR* was then calculated before [(n) CT scan vs (n-1) CT scan] and during [(n+1) CT scan vs (n) CT scan] for the PD-1 inhibitor cohort. The algorithm for *TGR* calculation is publicly available online.

<https://github.com/chferte/TumorGrowthRate/blob/master/TGR_calculator.R>

*Supplementary 2.* TABLE S1: Association between the genetic status and HPD for patients with non-small cell lung cancer treated with immunotherapy (n, %).

| Non-small cell lung cancer | Non-HPD (n=101) | HPD (n=13) | P |
| --- | --- | --- | --- |
| KRAS mutation |  |  | 0.063 |
| Mutated type | 16 (20.3) | 6 (50.0) |  |
| Wild type | 63 (79.7) | 6 (50.0) |  |
| Missing | 22 | 1 |  |
| EGFR mutation |  |  | 0.231 |
| Mutated type | 41 (51.9) | 4 (33.3) |  |
| Wild type | 38 (48.1) | 8 (66.7) |  |
| Missing | 22 | 1 |  |
